# Microglia phenotypes are associated with subregional patterns of concomitant tau, amyloid-β and α-synuclein pathologies in the hippocampus of patients with Alzheimer’s disease and dementia with Lewy bodies

**DOI:** 10.1186/s40478-022-01342-7

**Published:** 2022-03-16

**Authors:** Sonja Fixemer, Corrado Ameli, Gaël Hammer, Luis Salamanca, Oihane Uriarte Huarte, Chantal Schwartz, Jean-Jacques Gérardy, Naguib Mechawar, Alexander Skupin, Michel Mittelbronn, David S. Bouvier

**Affiliations:** 1 Luxembourg Centre for Systems Biomedicine (LCSB), University of Luxembourg, Belval, Luxembourg; 2 Luxembourg Center of Neuropathology (LCNP), Dudelange, Luxembourg; 3 Laboratoire National de Santé (LNS), National Center of Pathology (NCP), Dudelange, Luxembourg; 4 Swiss Data Science Center, ETH Zürich, Zürich, Switzerland; 5 Department of Psychiatry, Douglas Mental Health University Institute, McGill University, Montreal, QC Canada; 6 Department of Oncology (DONC), Luxembourg Institute of Health (LIH), Strassen, Luxembourg; 7 Department of Life Sciences and Medicine (DLSM), University of Luxembourg, Esch-sur-Alzette, Luxembourg; 8 Faculty of Science, Technology and Medicine (FSTM), University of Luxembourg, Esch-sur-Alzette, Luxembourg

**Keywords:** Alzheimer’s disease, Dementia with Lewy Bodies, Hippocampus, Microglia, Amyloid-β, Hyperphosphorylated tau, Phosphorylated α-synuclein

## Abstract

The cellular alterations of the hippocampus lead to memory decline, a shared symptom between Alzheimer’s disease (AD) and dementia with Lewy Bodies (DLB) patients. However, the subregional deterioration pattern of the hippocampus differs between AD and DLB with the CA1 subfield being more severely affected in AD. The activation of microglia, the brain immune cells, could play a role in its selective volume loss. How subregional microglia populations vary within AD or DLB and across these conditions remains poorly understood. Furthermore, how the nature of the hippocampal local pathological imprint is associated with microglia responses needs to be elucidated. To this purpose, we employed an automated pipeline for analysis of 3D confocal microscopy images to assess CA1, CA3 and DG/CA4 subfields microglia responses in post-mortem hippocampal samples from late-onset AD (*n* = 10), DLB (*n* = 8) and age-matched control (CTL) (*n* = 11) individuals. In parallel, we performed volumetric analyses of hyperphosphorylated tau (pTau), amyloid-β (Aβ) and phosphorylated α-synuclein (pSyn) loads. For each of the 32,447 extracted microglia, 16 morphological features were measured to classify them into seven distinct morphological clusters. Our results show similar alterations of microglial morphological features and clusters in AD and DLB, but with more prominent changes in AD. We identified two distinct microglia clusters enriched in disease conditions and particularly increased in CA1 and DG/CA4 of AD and CA3 of DLB. Our study confirms frequent concomitance of pTau, Aβ and pSyn loads across AD and DLB but reveals a specific subregional pattern for each type of pathology, along with a generally increased severity in AD. Furthermore, pTau and pSyn loads were highly correlated across subregions and conditions. We uncovered tight associations between microglial changes and the subfield pathological imprint. Our findings suggest that combinations and severity of subregional pTau, Aβ and pSyn pathologies transform local microglia phenotypic composition in the hippocampus. The high burdens of pTau and pSyn associated with increased microglial alterations could be a factor in CA1 vulnerability in AD.

**Supplementary Information:**

The online version contains supplementary material available at 10.1186/s40478-022-01342-7.

## Introduction

The decline of memory is a shared symptom in age-related neurodegenerative diseases [24]. The atrophy of the hippocampus, essential for memory formation and consolidation [9, 13, 49], is associated with memory impairment in normal ageing [34] and is predictive of Alzheimer’s disease (AD) and associated dementia [3, 26]. The hippocampus is one of the most severely affected brain regions in AD and dementia with Lewy Bodies (DLB) [4, 20, 28, 64] but the causes of its vulnerability are still poorly understood. The hippocampal architecture has been widely studied for its tri-synaptic loop circuitry and its composition in five distinct subfields, the *dentate gyrus* (DG), as well as the four *Cornu ammonis* (CA) fields [8] dedicated to specific types or sequences of memory processes [18, 82, 94]. The subregional pattern of atrophy of the hippocampus in AD is distinct from other groups of neurodegenerative conditions. The severe loss of volume in CA1 serves as an anatomical correlate in imaging to classify patients, CA1 being more preserved in patients with DLB compared to AD [1, 3, 40, 43, 53, 74, 96]. Understanding the molecular and cellular mechanisms that lead to such a subregional vulnerability in the AD hippocampus compared to a milder and more homogenous deterioration in DLB could unveil personalized therapeutic strategies to alleviate the progression of memory decline.

Microglia, the innate immune cells of the CNS, regulate homeostasis by clearing pathogens, cell debris and dying cells [55]. Microglia are constantly surveying their micro-environment with their moving ramifications and react quickly to insults by adopting an activated profile associated with a decrease of branches and an amoeboid form [79]. They are involved in numerous responses such as the secretion of anti- and pro-inflammatory molecules, and phagocytosis, that could be protective or detrimental by accelerating neuronal deterioration depending on the context [78]. Genome-wide association studies (GWAS) identified AD onset risk loci that are associated with genes involved in microglia physiology and responses, such as *CR1* (complement receptor type 1), *SPI1* (transcription factor PU.1), *TREM2* (triggering receptor expressed on myeloid cells 2) and *CD33* [61]. New technologies such as single cell RNA-sequencing, have redefined the roles and phenotypes of microglia in brain disorders and unveiled a complex regional and temporal heterogeneity of their signatures across mouse models and neurodegenerative conditions in human [7, 56, 69, 76, 90]. PET imaging correlated microglia activation with hippocampal volume loss [25] but if and how microglia play a role in the subregional deterioration pattern of AD is poorly understood. Furthermore, some studies have shown differences in microglia responses between AD and DLB [2, 6, 16, 52, 86, 87], DLB microglia seemingly less altered and responsive in numerous brain regions. How their subregional responses within the hippocampus differ from AD remains unclear. Additionally, microglia responses have been associated with misfolded protein pathologies in cell cultures and monogenic rodent models [36, 48, 51, 67, 77, 83, 95] but AD and DLB samples often show overlapping pathologies, extracellular senile plaques made of amyloid-β (Aβ) peptides, intracellular neurofibrillary tangles (NFTs) built from hyperphosphorylated tau (pTau) and intracellular aggregation of phosphorylated α-synuclein (pSyn) such as Lewy bodies [47], with different gradients of severity [12, 59, 60, 88]. The association between microglia phenotypes and mixed-pathological contexts still needs to be clarified.

In this study, we have investigated how microglia responses differ in AD and DLB cases across the CA1, CA3 and DG/CA4 subfields of the hippocampus and their relationship with pTau, Aβ and pSyn pathologies patterns. To this purpose, we immunostained thick and consecutive sections with Iba1, pTau, Aβ, or pSyn antibodies and employed high-content 3D confocal microscopy combined with image analysis tools such as our Microglia and Immune Cells Morphologies Analyser and Classifier (MIC-MAC) pipeline [75] on a collection of neuropathologically diagnosed post-mortem AD and DLB cases, and age-matched control (CTL) samples. Our approach allowed a very detailed and 3D understanding of microglia changes at the individual level in the various subfields of the hippocampus and highlighted their local association with concomitant pTau, Aβ and pSyn loads. We report for the first time the relation between the remodelling of local microglia population and the subregional pattern, the co-occurrence and severity of pTau, Aβ and pSyn pathologies across AD and DLB.

## Materials and methods

### Human brain samples and processing

All experiments involving human tissues were conducted in accordance with the guidelines approved by the Ethics Board of the Douglas Bell Canada Brain Bank (Douglas Mental Health University Institute, Montréal, QC, Canada) and the Ethic Panel of the University of Luxembourg (ERP 16-037 and 21-009). All pseudonymized autopsy brain samples were provided by the Douglas Bell Canada Brain Bank (formalin samples) and the Luxembourg Brain Bank (formalin-fixed paraffin-embedded (FFPE) samples) (see Table 1). Hippocampal samples (median part of the hippocampus, between the uncus and *corpus geniculatum laterale*) were dissected from cases of neuropathologically confirmed AD and DLB, as well as from age-matched CTLs. Samples were evaluated regarding Aβ plaques, NFTs and α-synuclein and assessed according to Braak, ABC staging [12, 63] and Mc Keith staging criteria [59] by an affiliated neuropathologist of the Douglas Bell Canada Brain Bank. This study comprises age-matched CTLs with no history of dementia. However, some CTL cases showed low levels of neuropathological abnormalities such as AD pathology. Brain samples used for the volumetric study were preserved in 10% formalin until processing as indicated in Table 1.

**Table 1:** Demographic and neuropathological data of human samples

Diagnosis	Case	Sex	Age (years)	PMD (hours)	Disease score	Reported concomitant misfolded protein pathologies*
*CTL*						
	1	F	86	5.7	/	/
	2	F	80	17.5	/	/
	3	F	89	23.6	/	Some senile plaques and NFTs
	4	M	89	32.2	/	/
	5	M	80	13.0	/	Some senile plaques
	6	F	83	35.7	/	/
	7	F	95	23.7	/	/
	8	M	85	26.7	/	/
	9	M	83	16.8	/	/
	10	M	89	31.9	/	A1B1C1
	11	F	55	21.1	/	/
*DLB*						
	12	M	70	24.5	Neo-cortical diffuse	Senile plaques and NFTs
	13	M	89	17.0	Limbic transitional	Mixed AD
	14	M	75	21.0	Neo-cortical	Senile plaques and NFTs
	15	M	64	17.2	Neo-cortical	Senile plaques and NFTs
	16	F	83	17.7	Neo-cortical	Senile plaques and NFTs
	17	M	82	22.0	Neo-cortical diffuse	/
	18	F	81	8.6	Neo-cortical diffuse	/
	19	F	74	27.6	Neo-cortical	A2B2C0
	20	M	91	85.0	Neo-cortical diffuse	Braak III/IV
*AD*						
	21	M	80	15.5	Braak IV	/
	22	M	82	11.5	Braak IV	/
	23	M	85	31.1	A2B3C2	/
	24	F	83	25.0	A2B3C2	/
	25	M	91	25.0	A2B3C2	/
	26	F	96	26.5	Braak IV/V	/
	27	M	87	21.7	A2B3C2	/
	28	M	90	26.1	A3B2C3	/
	29	F	87	17.0	A1B2C2	/
	30	M	90	32.0	A2B2C3	/

*AD* Alzheimer’s disease, *CTLs* age-matched controls, *DLB* Dementia with Lewy Bodies, *NFTs* neurofibrillary tangles, *PMD* post-mortem delay

*After neuropathological examination

#### Post-mortem case description

The description of the samples is detailed in Table 1. We ran our image-based analysis on age-matched CTLs (*n* = 11), with no or low levels of neuropathological alterations as well as on neuropathologically confirmed AD (A1B2C2 to severe stages A2B3C2 or Braak IV) (*n* = 10) and DLB (limbic transitional to neocortical diffuse) (*n* = 8) cases often bearing a certain amount of AD pathologies. The median age at death and average post-mortem delay (PMD) did not differ significantly between groups and are 83.1 years and 22.6 h for CTLs; 87.1 years and 23.1 h for AD; and 77.3 years and 19.4 h for DLB. Sex representation is slightly unbalanced with female cases being respectively 54.5% for CTLs, 30% for AD and 37.5% for DLB. Average time spent in fixative was also approximatively similar for specimens of all groups (CTLs, 14.7 years; AD, 11.1 years and DLB, 12.2 years).

#### Immunohistochemistry

The FFPE sample from the Luxembourg Brain Bank was used to validate pSyn, 4G8 and AT8 antibodies on 3 µm sections. Briefly, sections were processed by an automated immunostainer (Omnis Immunostainer, Agilent, Glostrup, Denmark) with primary antibodies 4G8, AT8 or against PS129-synuclein. For all three primary antibodies, heat retrieval was performed with the Envision Flex Tris low pH buffer (Agilent) for 30 min at 97 °C on board, primary antibodies were incubated 20 min at room temperature (RT), and detection was performed with the Envision Flex Detection kit (Agilent) based on the DAB/HRP substrate system. The sections were counterstained with hematoxylin.

The formalin hippocampal samples were washed in phosphate buffer saline (PBS), cryo-preserved in 30% sucrose in PBS for 36 h approximately and embedded in an M-1 embedding matrix (Thermo scientific, USA. Then, they were cut into 80 to 100 µm thick slices on a sliding freezing microtome (LeicaSM2010R) and preserved at − 20 °C in a cryoprotectant solution [Ethylene glycol (30%), and glycerol (30%) in 0.05 M phosphate buffer (PB, pH 7.4)]. Immunostainings were performed as previously described [10, 70] without antigen retrieval. In brief, after an overnight UV irradiation (UV lamp Ushio, 30 Watt) which reduces the autofluorescence of the human fixed samples [10, 70] sections were permeabilized for 30 min with 0.5% Triton-X 100 in PBS 1X. Subsequently, free-floating sections were incubated for 2 h with a blocking solution (0.5% Triton-X 100 and 2% horse serum in PBS 1X) at RT and incubated with primary antibodies in blocking solution for 72 h at 4 °C. Sections were then washed three times for 10 min in PBS 1X and subsequently incubated in 0.5% triton-X 100/PBS 1X at RT for 2 h with fluorophore coupled secondary antibodies. Sections were finally washed twice for 10 min in 0.1 M PB before mounting on glass slides using ProLong Gold Antifade reagent (Invitrogen). For analyzing the co-distribution of pTau and pSyn inclusions and for validating the 11A5 antibody staining, sections were incubated for an additional 20 min with the fluorescent DNA dye DRAQ7™ (1:100, Cell Signaling #7406) at RT and washed twice for 10 min in 0.1 M PB (pH 7.4) before mounting. See Tables 2 and 3 for references for primary and secondary antibodies. The immunostainings for Iba1, AT8, 4G8 or 11A5 antibodies that served for the analysis were done on consecutive slices. To improve homogeneity in the volumetric quantification analysis, samples were stained following the same combination of primary and secondary antibodies (respectively AT8 with anti-mouse Alexa Fluor 647, 4G8 with anti-mouse Alexa Fluor 488 and 11A5 with anti-mouse Alexa Fluor 488).

**Table 2:** Primary antibodies

Target	Epitope	Clone	Host species	Dilution	Manufacturer	Reference	RRID
pSyn	P-Ser129	11A5	Mouse (M)	1:500	Prothena Biosciences*	NA	NA
pSyn	P-Ser129	81A	Mouse (M)	1:200	Millipore	MABN826	AB_2904158
pSyn	P-Ser129	EP1536Y	Rabbit (M)	1:200	Abcam	ab51253	AB_869973
Aβ	AA17-24	4G8	Mouse (M)	1:200	BioLegend	800712	AB_2734548
Iba1/AIF-1	C-term	NA	Rabbit (P)	1:500	Wako	019-19741	AB_839504
NF-H	Met1-Ala380	NA	Goat (P)	1:500	Biotechne	AF3108	AB_2149640
pTau	Ser202, Thr205	AT8	Mouse (M)	1:500	Thermofisher	MN1020	AB_223647
pTau	Ser422	NA	Rabbit (P)	1:150	Thermofisher	44-764G	AB_1502115

*M* monoclonal, *NA* not available, *P* polyclonal

*Provided by Dr. Manuel Buttini

**Table 3:** Secondary antibodies

Fluorophore	Host	Reactivity	Isotype	Dilution	Manufacturer	Reference	RRID
Alexa Fluor 488	Donkey	Rabbit	IgG (H + L)	1:300	Jackson	711-545-152	AB_2313584
Alexa Fluor 488	Donkey	Mouse	IgG (H + L)	1:400	Jackson	715-545-150	AB_2340846
Alexa Fluor 488	Donkey	Goat	IgG (H + L)	1:400	Jackson	705-545-147	AB_2336933
Alexa Fluor 555	Donkey	Rabbit	IgG (H + L)	1:400	Invitrogen	#A-31572	AB_162543
Alexa Fluor 647	Donkey	Mouse	IgG (H + L)	1:300	Jackson	715-605-150	AB_2340862
Alexa Fluor 647	Donkey	Rabbit	IgG (H + L)	1:300	Jackson	711-605-152	AB_2492288

### Image acquisition and analysis

#### 2D image acquisition

All brightfield images of the FFPE DAB and hematoxylin-stained sections were captured by a light Leica brightfield DM2000 LED microscope (Leica Microsystems) with 20× and 40× air objectives and a Leica DMC2900 camera (Leica Microsystems).

#### 3D image acquisition

All confocal images for the MIC-MAC 2.0 analysis and for the quantification of pTau, Aβ and pSyn loads, 348 in total, were captured on a Zeiss LSM 710 with a 20× air objective. Some qualitative images were captured on a LSM 800 confocal system. 3D tile scans (z-steps of 1 µm) of hippocampal subfields from age-matched CTLs, DLB and AD patients were stitched via Zeiss or Imaris Stitcher and visualized with Imaris 9.5 and 9.6 (Oxford Instrument). 3D stacks of Iba1 for microglia morphology and of pTau, Aβ and pSyn inclusions were acquired on serial sections in the anatomically defined regions of interest representative of the entire layered structure of the CA1, CA3 and DG/CA4: from *Stratum oriens* to *Stratum moleculare* for CA1 and CA3, and of the DG including the hilus (CA4) as indicated in Additional file 1: Fig. S1, online resource.

#### MIC-MAC 2.0

Microglia and Immune Cells Morphologies Analyser and Classifier (MIC-MAC) 2.0 is a pipeline that allows for automatic segmentation of microglia and immune cells from fluorescent microscopy 3D acquisition. With respect to the previous version [75], automation and time performance have been improved. Source code and training samples are available on: https://doi.org/10.17881/w2d6-4934.

##### New automated segmentation of microglia

**Fig. 1:**
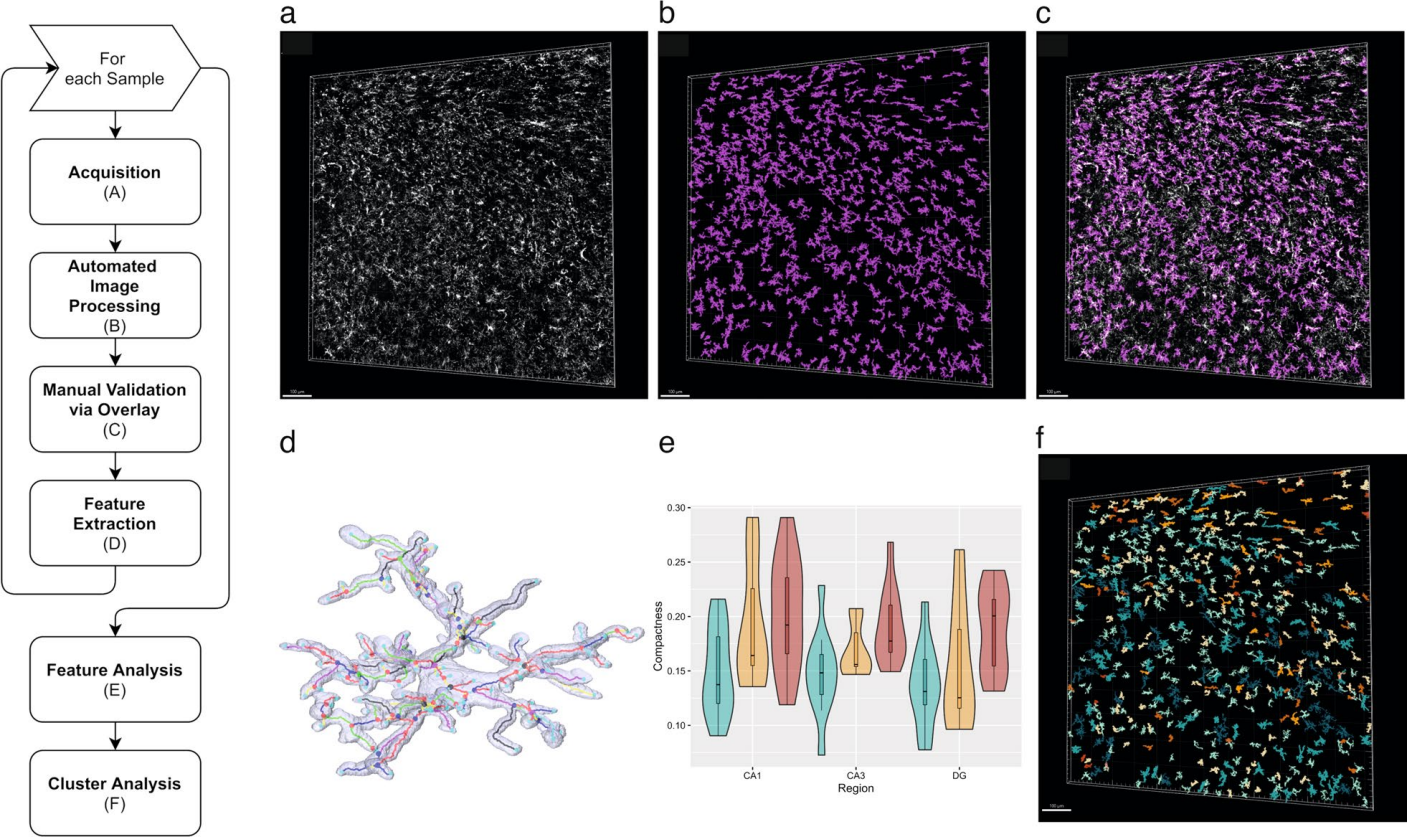
MIC-MAC 2.0 analysis workflow. MIC-MAC 2.0 pipeline with illustrations of acquisition (**a**), automated image processing (**b**), manual validation via overlay (**c**), feature extraction (**d**), feature analysis (**e**) and cluster analysis (**f**). **a–c** Original Iba1 staining of microglia is represented in white, segmented microglia cells in magenta. **d** Microglia 3D geometrical features are based on nodes (connection points) and edges (branches). **f** Spatial cluster colour coded overlay. Scale bars in **a–c**, **f** = 100 µm

The image processing pipeline of MIC-MAC 2.0 is fully automated. While the previous version was based on a semi-supervised approach, meaning that a portion of the acquisition was needed to be labelled as positive signal or background, the new version automatically segments structures of interest by tuning parameters related to the image processing pipeline. A flowchart of the image processing pipeline is presented in Fig. 1. The algorithm works by elaborating the 2D images of the 3D stacks one by one. First, a noise filtering is applied [22] with sigma = 25, followed by a background subtraction phase made with a Gaussian filter (sigma = [10–50]). After an intensity adjustment [matlab::imadjust], the image is deconvolved by using the Lucy-Richardson method [50, 71]. Finally, positive pixels are detected via global thresholding by defining a parameter for the threshold t. The only two parameters that have been changed throughout this phase are sigma from the Gaussian filter and t from the global thresholding. The parameters have been tuned accordingly to the amount of noise present in the image, which can change due to numerous factors (e.g., microscope parameterization, tissue conservation and antibody penetration). All morphologies of microglia were further validated by overlaying the segmented microglia object on the original 3D stack with the Iba1 staining.

##### Feature extraction and selection

Automated morphological feature extraction is performed at individual cell resolution [75]. Out of 62 morphological features and derived measures from the first version of MIC-MAC, we have selected a subset of 16 features by discarding highly correlated features (Pearson, higher than 80%). Among the 16 features are morphological and graph-based measures representing distinct aspects of the 3D morphology to measure fine changes and alterations. A detailed description of the 16 features can be found in Additional file 1: Fig. S3, online resource, with an illustration of prototypical minimum and maximum extreme values.

##### Artifact removal

Segmentation artifacts were automatically removed in three different steps: (1) By thresholding cell volume and keeping cells whose volume is between ~ 48,000 and ~ 216,000 µm^3^. The volumetric range was visually validated. (2) By considering the amount of cell body surface that is included in the border of the acquisition. This allows to exclude cells whose shape is incomplete and thus may alter the results. We specifically remove cells whose shared surface with the border is bigger than 7.5% of the total cell surface. This threshold has been manually tuned by visual validation. (3) By training a classifier based on morphological features. We trained a classifier (Boosted Tree–Ada Boost) [29] via manual labelling of artifacts and applied the classification to the whole dataset with k-fold validation to prevent overfitting. The removal via cell volume is performed right after the image processing segmentation in order to speed up the feature extraction phase. The latter two, are performed after the feature extraction phase right before the analysis. Feature and cluster analyses are performed on a total of 32,447 individual microglia morphologies.

##### Cluster analysis

All 16 selected features were initially normalized to the range 0 to 1 over the complete set of cells. We used Ward hierarchical clustering [93] on the set of 16 features. All the features have been normalized by range into a mapping from 0 to 1 renderings of cells belonging to specific clusters at different heights of the dendrogram. We assessed that *n* = 7 was the best set-up to segregate distinct morphological clusters. These differences were further validated by the statistical analysis of features distribution among clusters (Additional file 1: Fig. S4, online resource). Cells were subsequently projected on a Uniform Manifold Approximation and Projection (UMAP) [58] for visualization purpose.

#### Volumetric quantification of pTau, Aβ and pSyn pathologies

3D stacks were acquired following the same parameters for confocal laser scanning (magnification, resolution, laser power, offset and dwell time). All subfields were acquired from the same slide. All segmentations of the protein inclusions were done on Imaris 9.5.1 and 9.6 with the surface module following the same procedure for each marker (surface detail, absolute intensity, thresholds). After segmentation, all volumes of the resulting objects (3D reconstructions) were summed up. The final score (%) represents the percentage of the volume of the stack covered by the selected marker staining.

#### Statistical tests

For statistical comparisons between populations, we used Mann–Whitney U Test. For evaluating score and p-value of correlations we used Spearman Correlation. All tests were performed at a significance level of 5%. In accordance with the explorative nature of the analysis, no correction for multiple testing was necessary.

## Results

### Microglia morphological responses in hippocampal subfields of AD and DLB patients

To assess the subregional microglia responses that paralleled the local pathological context, we took advantage of the Microglia and Immune Cells Morphologies Analyser and Classifier (MIC-MAC) pipeline [75] that allows for semi-automated segmentation and classification of microglia cells based on their morphologies. Indeed, morphologies of microglia partially reflect their responses; surveying microglia are usually very ramified, whereas activated microglia present an amoeboid-like morphology. To improve time-consuming imaging and analytical processing, we implemented numerous changes to create a MIC-MAC 2.0 version which is now available for research as open source (https://doi.org/10.17881/w2d6-4934). MIC-MAC 2.0 allows a fully automated, more precise and faster segmentation of large 3D confocal stacks, an automated discard of the stack-border microglia that could create artifact structures (Fig. 1). For this study, we captured more than 43,000 Iba1-positive objects across 87 3D stacks, representative of each of the three hippocampal subfields for each case of our cohort. Before analysis, we found slight variations but no statistical differences on Iba1 staining density across subfields and conditions (Additional file 1: Fig. S2, online resource). After segmentation, MIC-MAC 2.0 imposed a volume threshold to select individual microglia but discard microglia accumulations that appeared as merged structures, such as the Aβ plaque associated microglia. In our hands, individual cellular boundaries of microglia forming accumulations around the plaques cannot be delimited even after stimulated emission depletion (STED) microscopy super-resolution imaging (data not shown), which justified their exclusion from our analysis. After the removal of artifactual objects, we obtained 32,447 individual cell 3D reconstructions validated for our analysis. MIC-MAC 2.0 retained 16 of the previous 62 morphological and graph-based features from MIC-MAC that were shown to recapitulate the complexity of microglia morphologies based on arborization and core parameters of 3D morphology such as volume or polarity (Additional file 1: Fig. S3, online resource).

### Variations of microglia morphological features across hippocampal subfields in AD and DLB cases

**Fig. 2:**
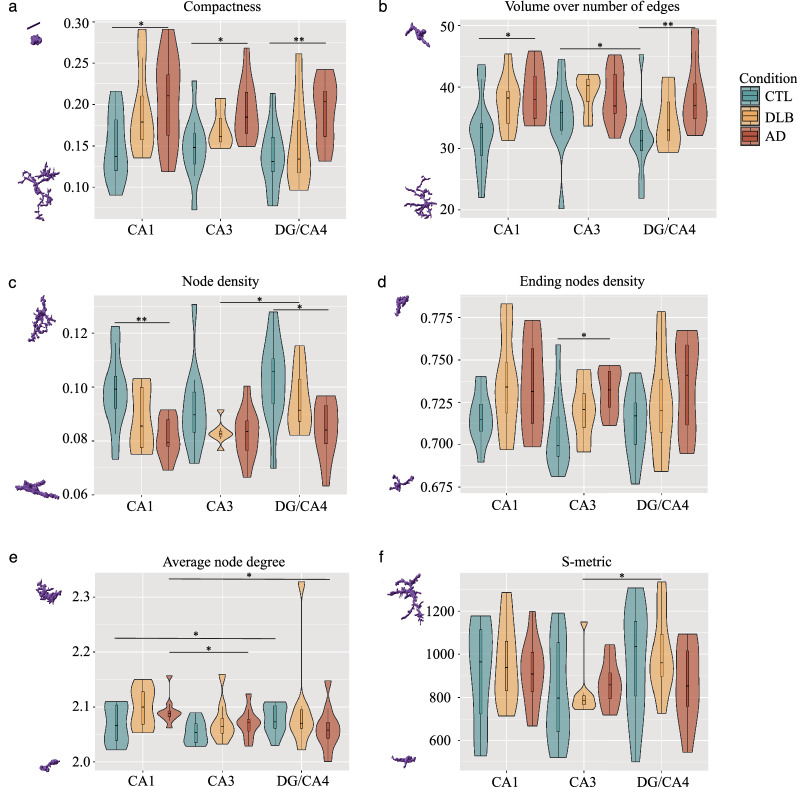
Subregional morphological alterations of microglia in the hippocampus of AD patients. Using MIC-MAC 2.0, we have extracted 16 geometrical features per cell of 32,447 individual microglia collected in CA1, CA3 and DG/CA4 subfields from AD, DLB and age-matched CTLs. None of the analyzed microglia morphological features was significantly changed in the DLB samples but follow a trend similar to the AD condition. All the following reported changes are observed in AD vs CTL samples. **a** Compactness is significantly increased in all three hippocampal subregions, **b** and volume over number of edges is significantly increased in CA1 and DG/CA4. (**c**) Node density significantly decreases in CA1 and DG/CA4, as does ending nodes density in CA3 (**d**). Two features, namely average node degree and s-metric present significant intra-condition regional changes (**e**, **f**). Wilcoxon–Mann–Whitney U-test *P* values are indicated in the graphs: *P < 0.05 and **P < 0.01. Scaled prototypic morphologies with highest and lowest value for the selected feature have been added on the left side. Scale bar = 20 µm

**Fig. 3:**
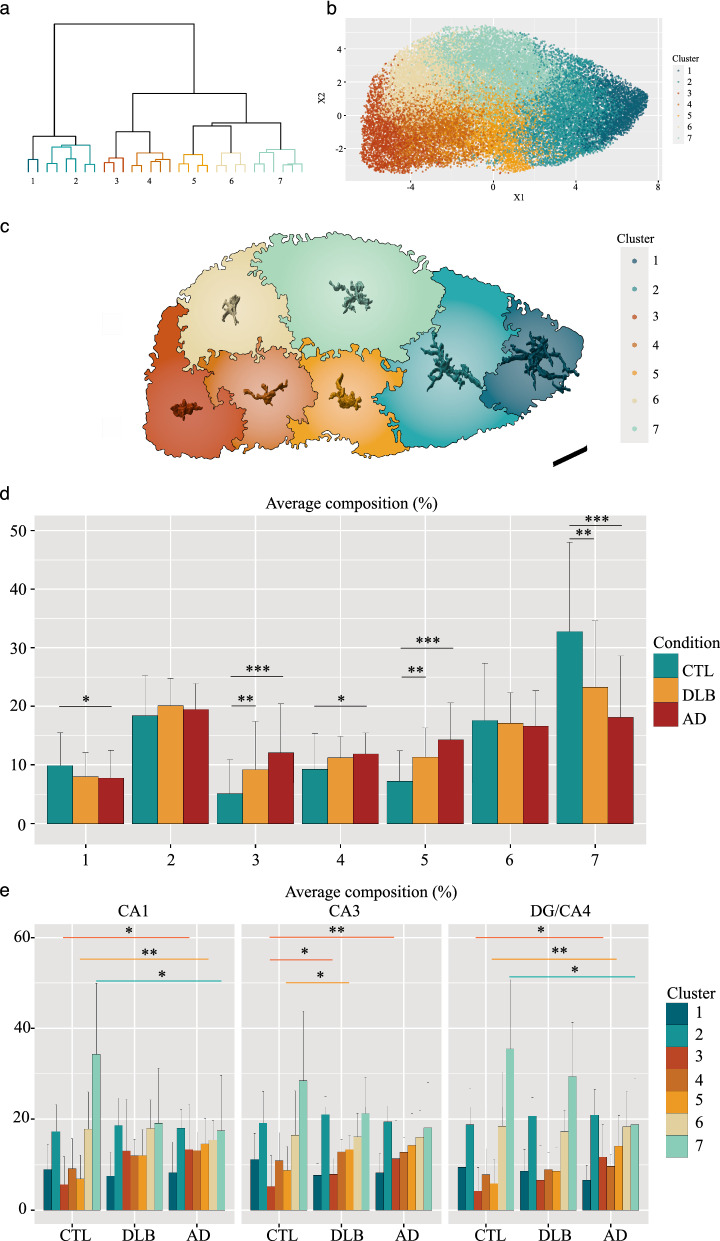
Microglia morphological cluster distribution varies according to the disease and the hippocampal subfield. Ward hierarchical clustering on the matrix of 32,447 microglia based on 16 features defines seven distinct morphological clusters. **a** Hierarchical clustering dendrogram. **b** Projection on a UMAP of the seven clusters where each of 32,447 individual microglia is represented by a dot. **c** Scaled prototypical morphologies of microglia associated to the seven clusters inside the UMAP. **d**, **e** Histograms showing the variation in abundance (average composition in %) of the seven clusters according to the condition (**d**) and across subfield per condition (**e**). Overall, clusters 3 and 5 are enriched in the disease conditions, whereas cluster 7 is depleted. Scale bar in **c** = 30 µm. Wilcoxon–Mann–Whitney U-test *P* values are indicated in the graphs: *P < 0.05; **P < 0.01 and ***P < 0.001

First, we measured the 16 morphological features of extracted microglia (list, definition and examples of their maximum and minimum prototypical structures in Additional file 1: Fig. S3, online resource) to assess the precise changes in their morphologies across subfields and conditions (Fig. 2). Interestingly, DLB values presented a similar trend to AD samples for almost all features and subfields when compared to CTLs, but without reaching significance. Among the 16 features, we found only four of them significantly different in AD compared to CTLs in at least one subfield. The compactness was the only feature significantly increased in all subfields in AD compared to CTLs (*P* < 0.05 for CA1 and CA3, *P* < 0.001 for DG/CA4) (Fig. 2a). High values for compactness are corresponding to amoeboid morphologies. Volume/number of edges significantly increased, and node density decreased in CA1 (both *P* < 0.05) and DG AD (respectively *P* < 0.05 and *P* < 0.01) (Fig. 2b, c). High values for volume/number of edges and low values for node density are matched with a simplified morphology with few or no branches. Ending nodes density increased in CA3 AD (*P* < 0.05) and indicates the enrichment of microglia with a low number of principal branches without secondary branches (Fig. 2d). Intra-condition, we found that the feature average node degree is significantly higher in CA1 AD than in CA3 and DG/CA4 AD *(P* < 0.05 and *P* < 0.05; Fig. 2e), s-metric and node density lower in CA3 than DG/CA4 in DLB (*P* < 0.05 Fig. 3f, and *P* < 0.05 Fig. 2c). Overall, compared to the CTLs microglia from the same subfield, CA1 AD microglia population adopted a morphology with higher compactness and loss of branching. AD DG/CA4 microglia followed a similar trend but to a lesser degree. AD CA3 microglia also showed a decrease in their complexity but with a distinct fine-tuning of their morphology.

### Microglia morphological clusters composition in the hippocampus AD and DLB patients

Feature analysis allowed us to dissect the fine details of microglia morphologies. To further characterize how microglia populations are remodeled across the hippocampal subfields in AD and DLB, we applied a cluster analysis to our matrix of morphological data. Briefly, we have selected seven clusters after a Ward hierarchical clustering based on a normalized set of the 16 features (Fig. 3a). The seven clusters showed distinct morphological patterns with specific characteristics and were distributed accordingly on an UMAP (Fig. 3b, c, Additional file 1: Fig. S4a–c, online resource). Briefly, clusters 1, 2 and 7 were composed by branched and complex cells but showed clear differences in their volume and polarity (high for cluster 1, intermediate for cluster 2 and low for cluster 7). Cluster 3 represented the most compact and smallest microglia. Clusters 4, 5 and 6 showed intermediate shapes mainly segregated by distinct features such as volume (higher in cluster 5), mean edge length (higher in cluster 4), and node density (higher in cluster 6) (Additional file 1: Fig. S4d–i, online resource). We then analyzed clusters relative abundances across conditions (Fig. 3d). We found a general decrease of two of the most ramified clusters, 1 and more severely of 7, in AD compared to CTLs (respectively *P* < 0.05 and *P* < 0.001) and of 7 in DLB (*P* < 0.05). Cluster 3 and 5 were enriched in both disease conditions compared to CTLs (cluster 3: AD *P* < 0.001, DLB *P* < 0.01) (cluster 5: AD *P* < 0.001, DLB *P* < 0.01). Clusters 2 and 6 stayed stable across conditions and cluster 4 was slightly but significantly increased in AD vs CTLs (*P* < 0.05). We then analyzed the changes of cluster composition by subfield across conditions (Fig. 3e). We reached significance for cluster 3 increase for all subfields in AD compared to CTLs (*P* < 0.05 in CA1; *P* < 0.01 in CA3 and *P* < 0.05 in DG/CA4) and in CA3 DLB *(P* < 0.05); for cluster 5 increase in CA1 AD, DG/CA4 AD (*P* < 0.01) and CA3 DLB *(P* < 0.05), and for cluster 7 decrease in CA1 and DG/CA4 AD (*P* < 0.05). These results show that microglia follow the same trends in AD and DLB cases compared to age-matched CTLs. However, they are less pronounced in DLB. At the subregional level, strong remodelling affects the CA1 and DG/CA4 microglia populations in AD and the ones of the CA3 in DLB. Additionally, the morphological cluster composition might highlight a microglia functional heterogeneity. Some clusters specifically changed depending on the subfield and condition. In particular clusters 3 and 5, two morphologically distinct groups, were enriched in disease conditions. Other clusters such as 2 and 6 appeared unaffected by the pathological context.

### Co-occurrence and severity patterns of pTau, Aβ and pSyn pathologies in the hippocampal subfields of AD and DLB samples

**Fig. 4:**
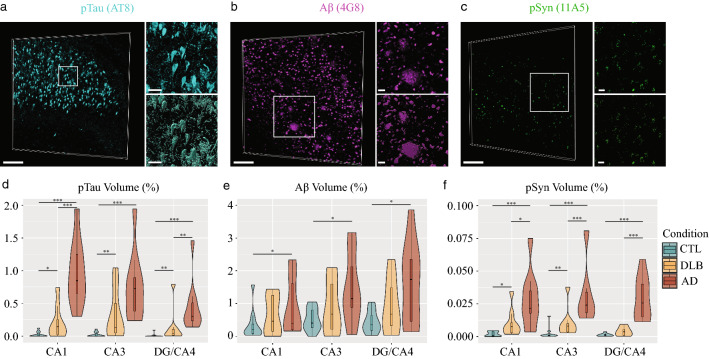
PTau, Aβ and pSyn loads show distinct disease-associated, subregional patterns in the hippocampus of AD and DLB patients. PTau, Aβ, and pSyn burdens were assessed via immunofluorescence staining (big panels and zoom in small upper panels) and volumetric analysis in CA1, CA3 and DG/CA4 of age-matched CTLs, AD and DLB patients. The staining signal was segmented (with Imaris 9.6) (small lower panels) and their volume measured from 3D acquisitions of each hippocampal subfield for each sample. **a** PHFs and NFTs of pTau (cyan) were stained with anti-AT8, example of an 80-year-old male AD patient (case 21). **b** Aβ plaques and subcellular inclusions (magenta) were stained with anti-4G8, example of a 90-year-old male AD patient (case 30). **c** PSyn inclusions, Lewy bodies and Lewy neurites (green) were stained with anti-11A5, example of a 64-year-old male DLB patient (case 15). Violin plots (with box plots) show that **d** PTau levels are significantly higher in the AD samples as compared to both DLB and CTLs in all three hippocampal subfields, but a significant increase was also observed in DLB samples compared to CTLs. **e** Aβ levels are significantly higher in AD samples in all three subfields compared to CTLs, whereas no significant differences are seen between DLB and both AD and CTLs. Aβ loads follow the same subregional pattern in DLB but with lower values. **f** PSyn loads are significantly increased in AD in all subfields, and in CA1 and CA3 in DLB compared to age-matched controls. PSyn volumes in all subfields are also significantly higher in AD samples compared to DLB. Wilcoxon–Mann–Whitney U-test *P* values are indicated in the graphs: *P < 0.05; **P < 0.01 and ***P < 0.001. Scale bars in **a**–**c** big panels = 200 µm, small panels = 30 µm

To assess how microglia changes were affected by the subregional pathological context, we then employed confocal microscopy to measure pTau, Aβ and pSyn loads across subfields and conditions in consecutive sections. The analysis of the fluorescent staining of AT8 for pTau and 4G8 for Aβ antibodies, which are typically used for neuropathology assessment, showed expected inclusions and labeled structures, i.e., typical paired helical filaments (PHF) and NFTs with AT8 [32], Aβ plaques and some amyloid precursor protein (APP) detected in neuronal soma with 4G8 [37] (Fig. 4a, b; Additional file 1: Fig. S5, online resource; Additional file 1: Fig. S6, online resource). The 11A5 pSyn staining revealed multiple types of inclusions, from Lewy neurite-like structures to intracellular granular staining (Fig. 4c; Additional file 1: Fig. S6, online resource). To validate the 11A5 antibody against PS129 pSyn, we immunostained 3 μm thick FFPE sections of the amygdala from a DLB case with 11A5, 81A (Millipore) and EP1536Y (Abcam) antibodies all directed against the same PS129 target. All antibodies revealed a similar pattern typical of pSyn pathology such as Lewy neurites, Lewy bodies and intracellular vacuolar aggregations in neurons (Additional file 1: Fig. S7a, online resource). We then immunostained thick sections of formalin-fixed subiculum-entorhinal cortex of the same case, with a combination of an anti-PS129 antibodies (11A5, 81A or EP1536Y), an antibody against pan-axonal neurofilament to detect neurons (NF-H) and DRAQ7™ to stain nuclei and analyzed the 3D confocal images (Additional file 1: Fig. S7b, online resource). Again, all PS129 antibodies gave a broadly similar staining with labeled structures resembling Lewy bodies and Lewy neurites found enclosed in neurons double-stained for NF-H and DRAQ7™. The 11A5-positive structures found in our cohort of AD and DLB samples resembled Lewy neurites and Lewy bodies but also revealed cellular inclusions and PHF-like structures, which were also seen with the 81A antibody (Additional file 1: Fig. S8, online resource).

After validation of the 11A5 antibody, we acquired representative 3D image stacks of AT8, 4G8 and 11A5 staining of the CA1, CA3 and DG/CA4 subfields for each case. The images were segmented to estimate the relative volume covered by the pathology of interest (Fig. 4a–c). We found variations in the pathological protein loads across samples and conditions that were generally compatible with the neuropathology staging of the samples. However, we observed all three pTau, Aβ, and pSyn pathologies in AD as well as in DLB cases. The majority of DLB cases were already described with partial AD pathologies and we found low to intermediate levels of pTau, Aβ, and pSyn accumulations (Fig. 4d–f). The AD group showed the highest levels for all pathologies, including pSyn, in the three hippocampal subfields. The CTLs showed no or low level of pTau staining and low to moderate Aβ labelling in neuronal-like soma and in few extra-cellular deposits. In our CTL group, we also observed very few pSyn inclusions in cells looking like pyramidal neurons but no Lewy bodies or Lewy neurite like structures.

More precisely, pTau burdens were significantly higher in all subfields in AD and DLB compared to CTLs (AD vs CTLs: *P* < 0.001, DLB vs CTLs: *P* < 0.05) and followed a similar subregional pattern across conditions with the highest loads found in CA1 and CA3 and lowest in DG/CA4. The severity of the pTau pathology was considerably higher with the most extreme values in CA1 AD, and DG/CA4 AD compared to DLB respective subfields (*P* < 0.001 in CA1; *P* < 0.05 in DG/CA4). We found a distinct subregional pattern of Aβ loads, again, conserved across the conditions with the highest Aβ burdens in DG/CA4 and CA3 and lowest in CA1. Only Aβ AD values were significantly higher than CTLs ones for all subfields (*P* < 0.05). The subregional distribution of pSyn loads appeared more uniform across subfields but with the highest loads found in CA1 and CA3 in AD and DLB. Still, the volume recovered by pSyn staining were very low compared to Aβ or pTau ones. We found statistical significances for all subfields compared to CTLs for AD (*P* < 0.001), and for CA1 (*P* < 0.05) and CA3 (*P* < 0.01) in DLB. Surprisingly, the pSyn burdens were much higher in AD than DLB samples for all subfields (*P* < 0.05 CA1, *P* < 0.001 CA3 and DG/CA4). Our data suggest that pTau, Aβ and pSyn pathologies follow a specific subregional hippocampal pattern that is shared across AD and DLB. However, burdens were more severe in AD, with CA1 AD particularly affected by pTau accumulations.

**Fig. 5:**
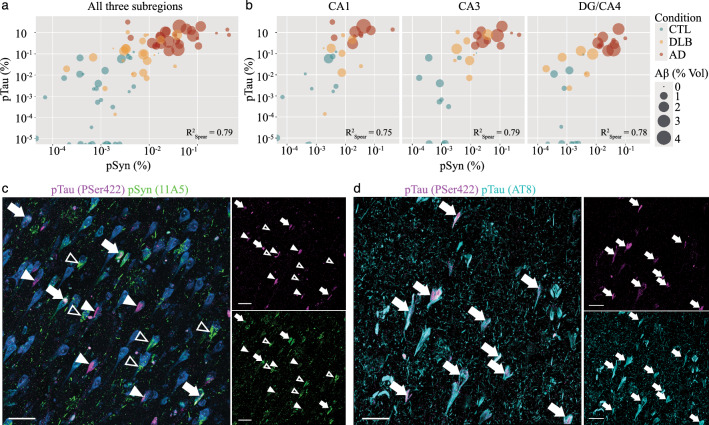
PTau and pSyn loads are strongly correlated across hippocampal subregions and conditions. The Spearman correlation bubble charts (log_2_ scale) display a strong positive correlation between pTau and pSyn loads across **a** conditions and subfields (R^2^_Spear_ = 0.79) and **b** at subregional level: CA1 (R^2^_Spear_ = 0.75), CA3 (R^2^_Spear_ = 0.79) and DG/CA4 (R^2^_Spear_ = 0.78). Corresponding Aβ loads are labeled by the size of the bubbles. Lower but positive correlation are observed between pTau and Aβ (R^2^_Spear_ = 0.30) and between pSyn and Aβ (R^2^_Spear_ = 0.26) across the three subregions. **c** The correlation between pTau and pSyn is also visible at cellular level with mainly mono-positive neurons for one or the other type of inclusion, but also some rare double-positive neurons that contain pTau and pSyn inclusions at the same time. CA1 from an 82-year-old male AD patient (case 22) immunostained for pSyn (mouse, 11A5, green), pTau (rabbit, PSer422, Thermofisher 44-764G, magenta) and fluorescent DNA dye DRAQ7™ (blue). Rare pyramidal neurons that co-express pSyn and pTau are indicated by full arrowheads. More commonly, pyramidal neurons are positive for either pTau (full triangles) or pSyn (empty triangles) inclusions. To facilitate visualization of mono- respectively double-positive neurons, the location of arrows and triangles is kept in all staining images. Nuclei of all cells are stained in blue. **d** Colocalization (arrowheads) of AT8 (mouse, cyan) and PSer422 (rabbit, magenta) is confirmed in the CA1 of a 90-year-old AD patient (case 28). Scale bars **c**, **d** 30 μm

Next, we examined the association between the three pathologies across subfields and conditions (Fig. 5a, b). Positive associations between pTau and Aβ and between pSyn and Aβ volumes of respectively R^2^_Spear_ = 0.30 and 0.26 were found across subfields and conditions but differ subregionally with superior values in the CA1 (respectively R^2^_Spear_ = 0.42 and 0.37 (*P* < 0.05)) and a loss of statistical significance in the CA3 and DG/CA4. However, we found a strong positive correlation between pTau and pSyn volumes across conditions (R^2^_Spear_ = 0.79; *P* < 0.001) that was preserved in all subfields (CA1: R^2^_Spear_ = 0.75; CA3: R^2^_Spear_ = 0.79; DG/CA4: R^2^_Spear_ = 0.78; *P* < 0.001). To better define pTau and pSyn associations at the cellular level, we tested double staining of pTau with a PSer422 rabbit antibody to pair with the 11A5 mouse antibody and DRAQ7™ (Fig. 5c). We also confirmed that stainings with pTau PSer422 (rabbit) and AT8 (mouse) colocalized in a CA1 AD case (Fig. 5d). We observed a global co-distribution of pTau and pSyn in the same microenvironment, however, colocalizations in the same neuron were sparse and mono-stained neurons more common. Thus, the strong positive correlation between pTau and pSyn does not point out intertwined intracellular co-pathologies but rather simultaneous mono pTau or pSyn accumulations in neighbouring hippocampal neurons.

### Associations between morphological microglia changes and pTau, Aβ and pSyn loads in AD and DLB hippocampi

**Fig. 6:**
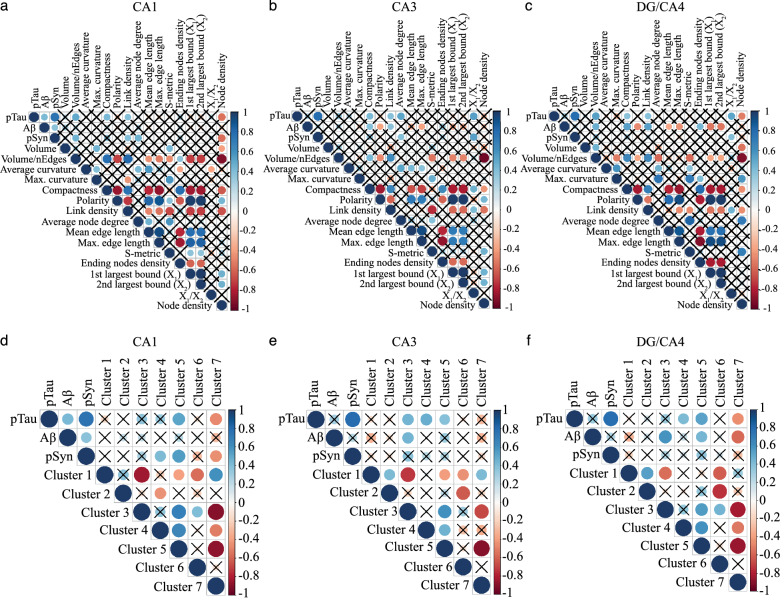
Microglial morphological features and clusters correlate with pTau, Aβ, and pSyn burdens in the hippocampus and follow a subregional pattern. Correlation plots between pathologies and microglial morphological features (**a**–**c**) and morphological clusters (**d**–**f**) in CA1, CA3 and DG/CA4 in CTL, AD and DLB conditions. A list and description of all morphological features with prototypic morphologies for lowest and highest value can be found in Additional file 1: Fig. S3 online resource). **a**–**c** Various microglia morphological changes are correlated to one or two AD and DLB pathologies in a subregion-dependent manner. **d**–**f** In a subfield-dependent manner, disease-enriched clusters 3 and 5 are positively correlated with one or two pathologies whereas disease-depleted cluster 7 present negative correlations. Statistically significant values (Spearman’s correlation) are indicated by a dot, the colour gives an estimation of the correlation (positive correlations in blue, negative correlations in red). Black crosses indicate unsignificant correlations

To evaluate the relationship between microglia responses and each pathology at the subregional level across conditions, we first evaluated the correlations (Spearman, statistically significant) between morphological features and pTau, Aβ or pSyn burdens across subfields (Fig. 6a–c). We found that the associations of pTau, Aβ and pSyn with features changed across subfields. In the CA1, pTau loads were correlated with some of the most altered features in DLB and AD, volume/number of edges, compactness, node density, and link density, a feature related to the complexity of the arborization. PSyn loads showed similar values of correlation than pTau ones with numerous features such as volume/number of edges, node density, and link density, but did not correlate with compactness and were positively associated with average node degree. Thus, we found a trend of correlations between the most altered features and a tandem of pTau and pSyn pathologies in the CA1 but an absence of statistically significant correlations with Aβ. In the CA3, some features were significantly associated with pTau (4) in tandem with Aβ (2) or pSyn (2), and 3 features uniquely to Aβ. In DG/CA4, a higher range of features were positively or negatively associated with Aβ (8), sometimes in tandem with pTau (3), with pTau alone (1) and pTau together with pSyn (1). The compactness, a feature statistically increased in AD, was associated with pTau loads in all subfields, but in tandem with Aβ only in the CA3 and DG/CA4. Volume/number of edges also changed in AD CA1 and DG/CA4, and showed an association with both pTau and pSyn in the CA1, and with pTau and Aβ in DG/CA4. Some features were specifically associated with one pathology such as node density with pTau or polarity, max and mean edge length, ending node and 1st and 2nd largest bound with Aβ. Overall, pTau in tandem with pSyn pathology seems to drive the most significant changes in the CA1 microglia, however Aβ, when highly expressed such as in DG/CA4, remains a potential dynamic modifier of microglia morphology.

At the cluster level, we also found a strong effect of the subregion on the associations between pathologies and clusters (Fig. 6d–f). In the CA1, we found positive correlations between cluster 5, the most branched of the disease-enriched cluster, and negative ones for cluster 7 composed by highly ramified cells, and the duo of pTau and pSyn loads, and a positive correlation between cluster 4 and pSyn. In the CA3, cluster 3 was positively correlated with the pair of pTau and Aβ loads, clusters 4 and 5 with pTau loads. In the DG/CA4, cluster 3 was associated with Aβ loads only and cluster 4 with pTau ones only, cluster 5 and 7 with the pair of pTau and Aβ loads.

This correlative analysis implies that each pathology impacts a specific set of morphological features of microglia but can also work in synergy with others to transform microglia toward clusters 3 and 5 morphology types. At the cluster level, the impact of pSyn is lower. PTau seems to be the stronger modifier in the CA1 and CA3 subregions, Aβ in the DG/CA4. Across subfield, pTau maintains a strong positive association with cluster 5 while cluster 3 is more correlated to Aβ. These data suggest that the severity, distribution but also co-occurrence of the three pathologies impact microglia phenotypic heterogeneity and condition their responses.

## Discussion

Our study offers a transversal and high-resolution description of the local morphological phenotypes of microglia in the hippocampal CA1, CA3 and DG/CA4 subfields across age-matched CTLs, AD and DLB conditions and their association with the subregional distribution and severity of the pTau, Aβ and pSyn pathologies. We have obtained high-content data about subregional patterning of pathologies in the human hippocampus in DLB and AD cases and unveiled its relationship with the remodelling of the microglia local population.

### Hippocampal microglia morphological remodelling is subregional and more severe in AD compared to DLB cases

Morphology of microglia only partially reflect their responses. However, visual description is commonly used to obtain a rapid readout of their alterations in CNS diseases. Indeed, the homeostatic microglia harbour a highly ramified profile in contrast to activated microglia that appear smaller, amoeboid-like with no or low number of branches. Here, we have analyzed 3D morphologies to characterize the local reorganization of microglia populations in hippocampal subregions of AD and DLB patients. We previously developed an AI pipeline called MIC-MAC, to classify and quantify morphologies of individual microglia labelled with the Iba1 marker in large 3D confocal image stacks of human or mouse post-mortem brains [75]. With MIC-MAC 2.0, we have significantly improved the automation and precision of the segmentation and added new features to discard artefacts upstream from the analysis. A caveat of our approach is that MIC-MAC 2.0 automatically discards all microglia accumulations, where the cells are so closely intermingled that our pipeline cannot separate them into individual cells after the segmentation process. In our hands, even super-resolution microscopy could not resolve the human microglia cellular boundaries in these accumulations. This argument strengthened our decision to focus on individual cells to overcome potential contaminations in our data but created a limitation and lowered the impact of Aβ on microglia changes in our analysis. However, we have created a substantial amount of information based on a sampling of 32,447 extracted individual microglia. By quantifying their 16 morphological features, our analysis highlighted disease and subregional particularities. When compared to their respective subfield in CTLs, we found statistical significances only for the AD condition. However, DLB variation trends always mimic AD ones showing that the presence of concomitant pathologies already initiated local remodelling but in a more subtle manner. Other reports found low to mild microglial changes in DLB samples with different approaches. Their conclusions were supported by 2D morphology analyses and quantifications of some activation markers in the hippocampus [6] and other brain areas such temporal lobe, the superior frontal gyrus [86] and the cerebral cortex [2]. However, our analysis implies that DLB hippocampal microglia are not so different from AD ones in their ability to respond and that their alterations are associated with the local level of severity of DLB- and AD-typical pathologies. High compactness was the only predictive feature for AD across subfields, strengthening the observation that microglia tend to become more amoeboid in AD, even when not attached to the plaques. At the morphological clusters level, in line with previous studies [6, 66], we show a decrease of the most branched and tortuous subgroups and an increase of amoeboid-like clusters significantly in AD. Interestingly, the low levels of the disease-increased clusters found in CTLs could be products of the ageing process or the onset of pathological mechanisms [14]. Our data also further suggest functional heterogeneity among microglia local subgroups. Indeed, we found that two morphological clusters stayed relatively stable over the conditions, while others were specifically depleted toward simplified morphologies. We have also uncovered two stereotypical morphologies enriched in AD and DLB, one amoeboid-like and one semi-ramified, which could reflect differential responses to pathological stressors [56]. PET imaging studies associated microglia activation with volume loss [25]. Here the increase of clusters 3 and 5 in AD and to a lower level in DLB samples is in line with this report and the more severe atrophy of the hippocampus observed in AD cases [1, 3, 40, 43, 53, 74, 96]. We also found a subregional remodelling of microglia populations that highlights more profound alterations in CA1 AD. Our quantitative analysis of pTau, Aβ and pSyn loads unveiled a tight and synergistic effect of the local misfolded protein pathologies with microglia changes.

### PTau, Aβ and pSyn burdens overlap and show specific subregional patterns in the hippocampus of AD and DLB cases

Here we report a high co-occurrence of the three types of pathologies studied, pTau, Aβ, and pSyn in the hippocampal subfields of AD and DLB cases. We have observed some low Aβ staining and pSyn accumulations but almost no pTau inclusions in our age-matched non-demented CTLs, in line with previous reports [21, 39, 42, 45, 54, 73]. With our 3D confocal approach, we found that pTau, Aβ and even pSyn burdens were all more severe in AD. The concomitance of pTau, Aβ, and pSyn loads is commonly observed in age-related neurodegenerative diseases [33] in both AD and DLB [60, 72] in numerous brain areas [17, 27, 81, 92]. However, our study revealed a complex tapestry of subregional pathological imprint in AD and DLB hippocampi, which each of the pathologies showing a specific subfield pattern. We found that pTau loads were more enriched toward CA1 and Aβ ones toward DG/CA4. PSyn loads were slightly higher in CA1 and CA3 but appeared more homogeneous across subfields. DLB samples presented intermediate values of pTau, Aβ and pSyn pathologies. Overall, our data are consistent with existing literature regarding the subregional distribution of pTau pathology in AD and DLB [11, 19], but slightly differ regarding Aβ subregional distribution [88] and pSyn higher loads in AD. The pSyn burden observed in the hippocampus across AD and DLB cases was very mild compared to Aβ and pTau ones. However, confocal microscopy allowed us to reveal frequent pSyn intracellular granular inclusions in cells identified as pyramidal neurons along with typical Lewy neurites and few Lewy bodies that might uncover an early sequence of pSyn pathology. Interestingly, we report stronger associations between pTau and pSyn burdens than between pTau or pSyn and Aβ loads. We found that the strong positive correlation between pTau and pSyn pathologies depended mainly on an exclusive distribution of pTau or pSyn inclusions in neighbouring hippocampal neurons rather than on a colocalization in the same cell, an observation that could suggest a systematic mechanistic association. However, we observed few double-positive hippocampal neurons carrying pTau and pSyn pathologies such as previously found in the subiculum [38], the amygdala [80] and parahippocampal gyri [35] in AD and DLB cases. In a previous study, the local co-distribution of NFTs and Lewy bodies was found more frequent in limbic areas than other brain parts [17]. Tau and synuclein proteins seem to be part of the same interactome in induced pluripotent stem cells (iPSC)-derived neuron models [89] but our observations contradict the idea of a stereotypic dual pathology in the AD and DLB hippocampus. How pSyn- and pTau-bearing neighbouring neurons co-inhabit the AD and DLB hippocampus and how the two pathologies coincide in few others remains to be understood. AD and DLB are complex diseases, and our study faces some limitations for generalizing our neuropathology findings such as the number of cases (29) included in our analysis. However, we did not evaluate the impact of TAR DNA binding protein of 43 kDa (TDP-43) pathology. Abnormal accumulations of TDP-43 in neurons were originally associated with frontotemporal lobar degeneration and amyotrophic lateral sclerosis. TDP-43 inclusions are now frequently reported in AD [62] and DLB + AD cases [65] and could play a substantial role in the hippocampal atrophy rate [41]. Finally, we have analyzed the medium part of the hippocampus, i.e., the body, between the uncus and *Corpus geniculatum laterale*, a region which is usually poorly described, contrary to the head at the uncus level or the tail close to the *Corpus geniculatum laterale*, both more regularly used for neuropathology diagnostic and research. We cannot exclude differences with previous reports as it has been shown that the hippocampus is molecularly and functionally diverse along its longitudinal axis [5, 85] and may be affected by different local levels of pathologies.

### Combinatory pattern of pTau, Aβ and pSyn loads and coinciding microglia responses shape the subregional deterioration of the hippocampus

Our study demonstrates that the combinatory pattern and the severity of pTau, Aβ and pSyn pathologies are associated with specific local microglia responses within the hippocampus. Each of pTau, Aβ and pSyn pathology seems to impact, often in tandem, the local microglia remodelling in AD and DLB hippocampi. We found fine-tuned associations with specific features or clusters that reflect the tight interdependence of microglia states with their microenvironment. Indeed, the correlation analysis showed that pTau and Aβ pathologies can be positively or negatively associated with a specific set of morphological features even if they also seem to work in synergy on some others. PSyn pathology appears to have a milder effect, here always paired with the pTau pathology. The morphological clusters 3 and 5 might be the selective products of some of these complex interactions, cluster 3 being more associated with Aβ loads and cluster 5 with pTau loads and could reflect molecularly distinct microglia states. Numerous microglia AD signatures, such as the disease-associated microglia [30, 44, 46], have been characterized in monogenic animal models mimicking Aβ pathology and were often observed in microglia surrounding Aβ plaques. If these signatures are fully or partially, regionally or sub-regionally recapitulated in human conditions still needs to be further characterized [15, 23, 57, 68, 84, 91]. Recently, Gerrits et al. [31] have defined by single-nuclei RNA sequencing that some signatures of microglia were selectively associated with Aβ (4G8), or with combined Aβ (4G8) and pTau (AT8) pathologies, in the occipital and occipitotemporal cortices of AD patients, but without investigating the possible impact of pSyn pathology. Our data support the idea of such a molecular microglia heterogeneity across the subfields of the hippocampus in AD and DLB. The complex pathological pattern of the human condition certainly represents a factor of divergence of microglia phenotypes between AD patients and monogenic rodent models. It calls for more multi-disciplinary and translational research before aiming for specific microglia signatures in the treatment of AD or DLB patients. However, if our comparison stands true at the scale of the brain, targeting microglia in DLB might be as promising as in AD.

## Conclusion

Our analysis suggests that the co-occurrence of the three pTau, Aβ and pSyn pathologies is frequent in the hippocampus of AD and DLB patients but with a severity largely increased in AD cases. Each pathology, pTau, Aβ and pSyn, seems to follow a specific subregional pattern, mainly conserved across AD and DLB. Combinations and severity of subregional misfolded proteins pathologies in the hippocampus transform the local microglia phenotypic composition. The high burdens of pTau and pSyn associated with increased microglial alterations could exacerbate the CA1 vulnerability in AD.

## Supplementary Information

**Additional file 1**: **Fig. S1**. Hippocampal subfields. Anatomically defined CA1, CA3 and DG/CA4 subfields of the hippocampus for pTau, Aβ, pSyn and Iba1 z-stack acquisitions. Hippocampal section of an 83-year-old female AD patient (case 24) with Aβ (4G8, green) and microglia (Iba1, magenta) stainings. Scale bar = 1000 µm

**Additional file 2**: **Fig. S2**. Iba1 density in hippocampal subfields of AD and DLB do not differ from age-matched CTLs. Iba1 volume (%) across conditions and hippocampal subfields

**Additional file 3**: **Fig. S3**. List and description of all morphological features with prototypic morphologies for lowest and highest feature values. Scale bars = 20 µm

**Additional file 4**: **Fig. S4**. Microglia morphological clusters are defined by morphological features. Projection on a UMAP where each of the 32,447 individual microglia is represented by a dot with distribution spectra for volume (**a**), compactness (**b**) and polarity (**c**). Violin plots showing the variations of volume (**d**), compactness (**e**), polarity (**f**), ending nodes density (**g**), link density (**h**) and mean edge length (**i**) in specific clusters

**Additional file 5**: **Fig. S5**. Validation of Aβ and pTau stainings on FFPE samples of a neuropathologically confirmed DLB case. 3 µm thick paraffin block sections from the temporal superior median gyrus and frontal median gyrus were obtained from the same 91-year-old male DLB patient (case 20) and were stained against Aβ (4G8, brown) and pTau (AT8, brown) based on the DAB/HRP substrate system with hematoxylin counterstaining. The lower rows represent a zoom of the indicated region in the upper row. Scale bars upper row = 100 µm and lower row = 50 µm

**Additional file 6**: **Fig. S6**. Confocal stainings of pTau, Aβ and pSyn across conditions and hippocampal subfields. 80–100 µm thick hippocampal sections were immunostained with the AT8 antibody against pTau (Ser202, Thr205) (cyan), the 4G8 antibody against Aβ (AA17-24) (magenta) and the 11A5 antibody against pSyn (Ser129) (green). The stainings show heterogenous distribution and types of inclusions across conditions and hippocampal subregions. Scale bars = 100 µm

**Additional file 7**: **Fig. 7**. Validation of pSyn stainings with three different antibodies on FFPE samples of a neuropathologically confirmed DLB case. 3 µm thick paraffin block sections from the amygdala and 80–100 µm thick sections from fixed hippocampus were obtained from the same 91-year-old male DLB patient (case 20) and were stained against pSyn using three different antibodies, namely 11A5, 81A and EP1536Y, that all three recognize the P-Ser-129 epitope. (**a**) Sections from paraffin blocks were stained against pSyn (11A4, 81A and EP1536Y; brown) based on the DAB/HRP substrate system. The sections were counterstained with hematoxylin. The lower rows represent a zoom of the indicated region in the upper row. (**b**) Thick sections from fixed samples were stained by immunofluorescence against pSyn (11A4, 81A and EP1536Y; green), neurofilaments (NF-H, magenta) and all nuclei (DRAQ7^TM^, blue). Scale bars in (**a**) upper row = 500 µm and lower row = 50 µm; (**b**) upper row = 100 µm and lower row = 10 µm

**Additional file 8**: **Fig. 8**. Confocal description of pSyn staining in a neuropathologically confirmed AD case. 80-100 µm thick sections from fixed hippocampus from a 90-year-old male AD patient (case 28) were immunostained against pSyn (11A5, 81A; green), neurofilaments (NF-H, magenta) and all nuclei (DRAQ7, blue). The upper row shows the hippocampus (stratum oriens at left bottom corner), and the lower row represents a zoom of the marked areas in the pyramidal layer. PSyn inclusions are present under various forms, including PHF-like (full triangle), Lewy neurite (empty triangle) and vacuolar aggregations (arrowhead). Scale bars upper row = 50 µm and lower row = 20 µm

## Data Availability

Source code and raw data are available on https://doi.org/10.17881/w2d6-4934.

## Abbreviations

Aβ: Amyloid-β
AD: Alzheimer’s disease
APP: Amyloid precursor protein
CA: *Cornu ammonis*
CTL: Control
DG: *Gyrus dentatus*
DLB: Dementia with Lewy Bodies
MIC-MAC: Microglia and immune cells morphologies analyser and classifier
NFTs: Neurofibrillary tangles
PMD: Post-mortem delay
pSyn: Phosphorylated α-synuclein
pTau: Hyperphosphorylated tau
UMAP: Uniform manifold approximation and projection
