# Supplementary figures and images for: Microglia phenotypes are associated with subregional patterns of concomitant tau, amyloid-β and α-synuclein pathologies in the hippocampus of patients with Alzheimer’s disease and dementia with Lewy bodies
Source: Acta Neuropathol Commun. 2022 Mar 16;10:36. doi: 10.1186/s40478-022-01342-7 (PMC8925098; doi:10.1186/s40478-022-01342-7)

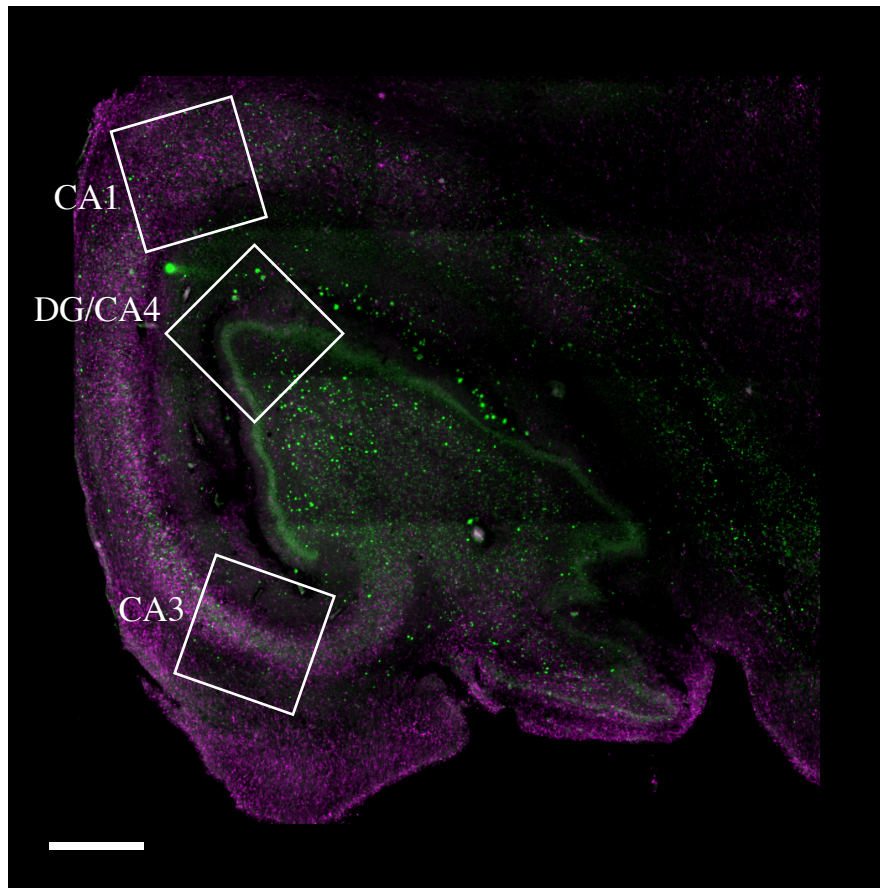

**Supplementary Fig. 1**

Supplement: Supplementary file 1 — Additional file 1: Fig. S1. Hippocampal subfields. Anatomically defined CA1, CA3 and DG/CA4 subfields of the hippocampus for pTau, Aβ, pSyn and Iba1 z-stack acquisitions. Hippocampal section of an 83-year-old female AD patient (case 24) with Aβ (4G8, green) and microglia (Iba1, magenta) stainings. Scale bar = 1000 µm [file 40478_2022_1342_MOESM1_ESM.pdf]

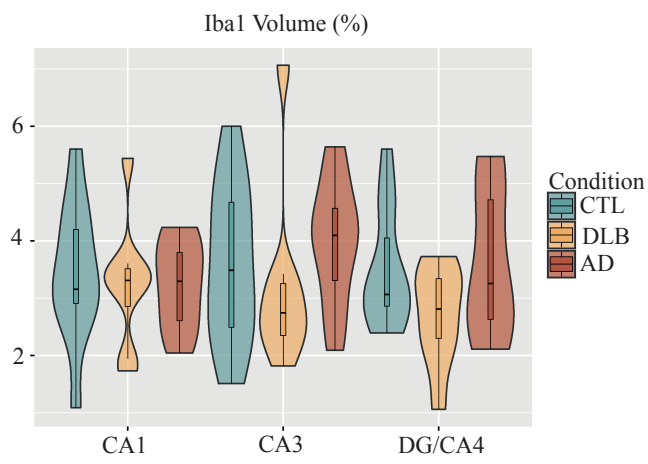

**Supplementary Fig. 2**

Supplement: Supplementary file 2 — Additional file 2: Fig. S2. Iba1 density in hippocampal subfields of AD and DLB do not differ from age-matched CTLs. Iba1 volume (%) across conditions and hippocampal subfields [file 40478_2022_1342_MOESM2_ESM.pdf]

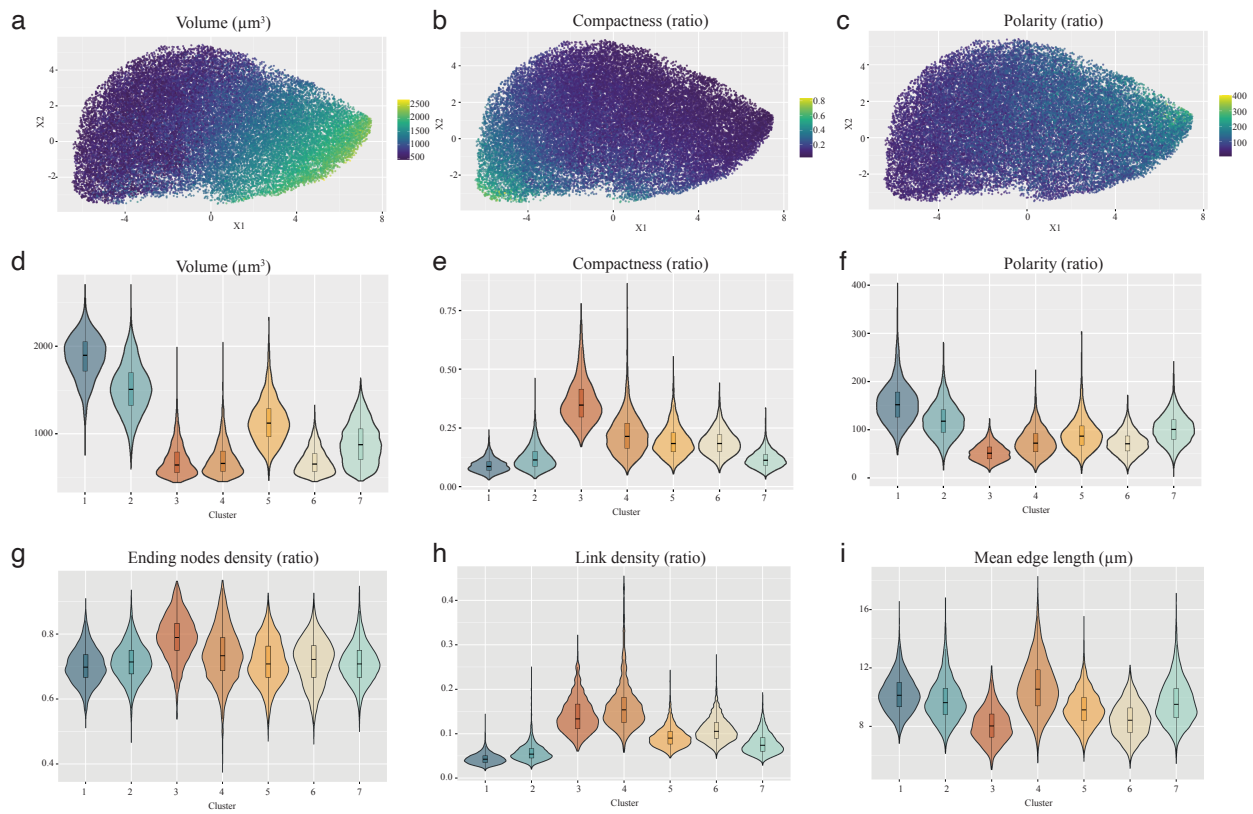

**Supplementary Fig. 4**

Supplement: Supplementary file 4 — Additional file 4: Fig. S4. Microglia morphological clusters are defined by morphological features. Projection on a UMAP where each of the 32,447 individual microglia is represented by a dot with distribution spectra for volume (a), compactness (b) and polarity (c). Violin plots showing the variations of volume (d), compactness (e), polarity (f), ending nodes density (g), link density (h) and mean edge length (i) in specific clusters [file 40478_2022_1342_MOESM4_ESM.pdf]

4G8

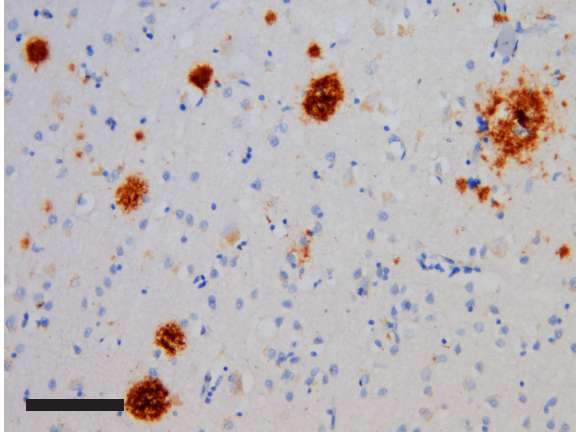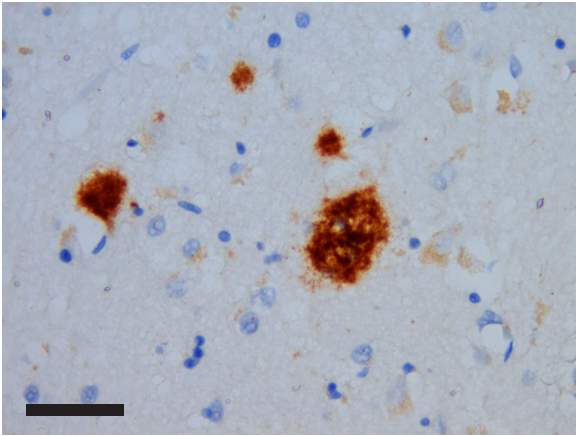

AT8

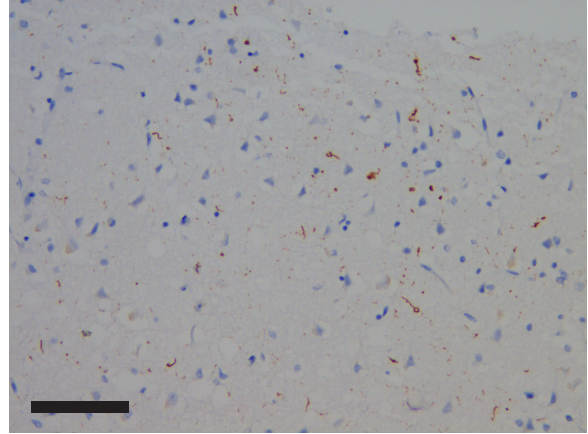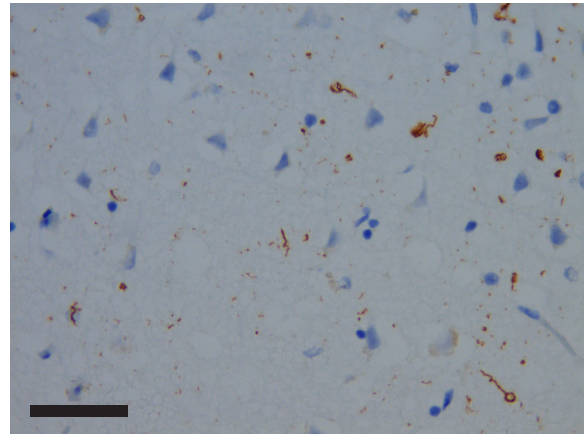

**Supplementary Fig. 5**

Supplement: Supplementary file 5 — Additional file 5: Fig. S5. Validation of Aβ and pTau stainings on FFPE samples of a neuropathologically confirmed DLB case. 3 µm thick paraffin block sections from the temporal superior median gyrus and frontal median gyrus were obtained from the same 91-year-old male DLB patient (case 20) and were stained against Aβ (4G8, brown) and pTau (AT8, brown) based on the DAB/HRP substrate system with hematoxylin counterstaining. The lower rows represent a zoom of the indicated region in the upper row. Scale bars upper row = 100 µm and lower row = 50 µm [file 40478_2022_1342_MOESM5_ESM.pdf]

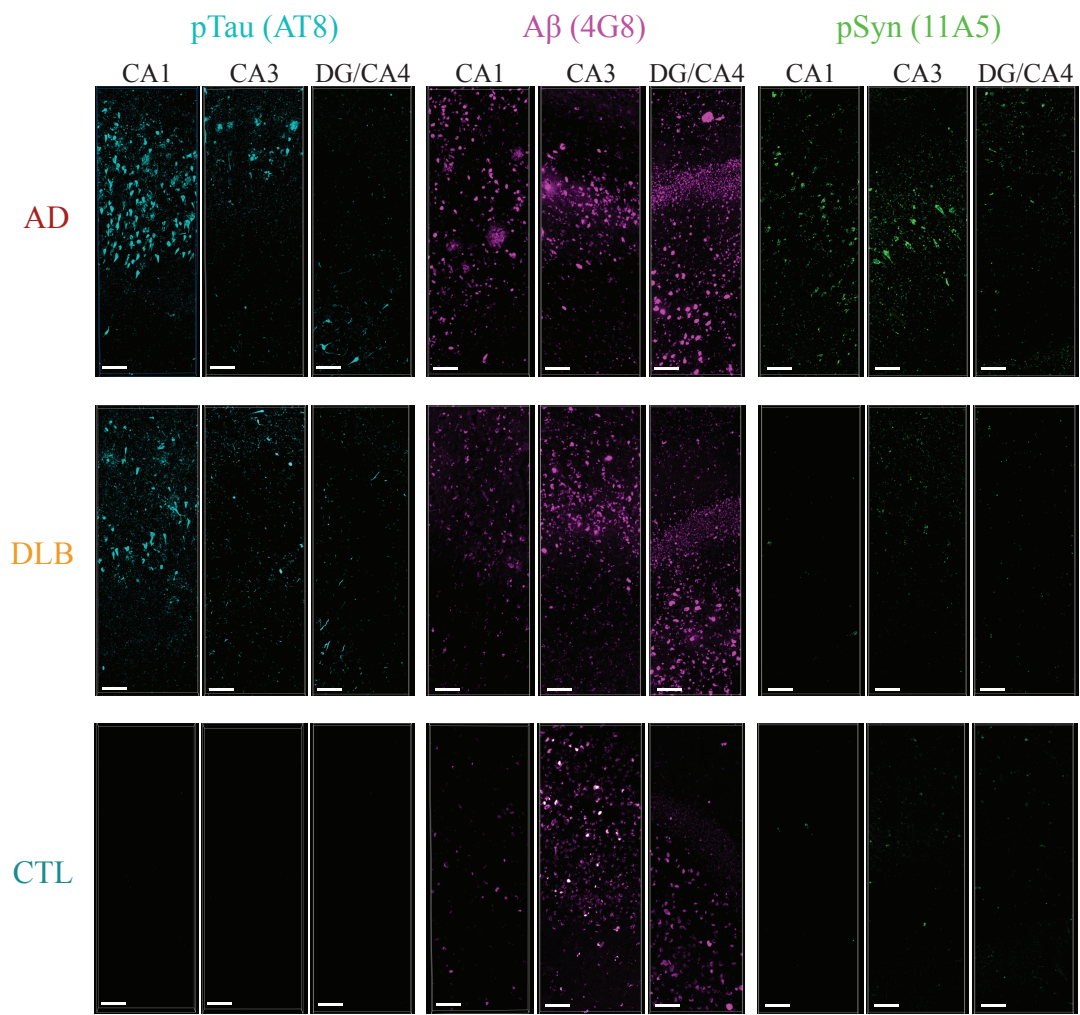

Supplementary Fig. 6

Supplement: Supplementary file 6 — Additional file 6: Fig. S6. Confocal stainings of pTau, Aβ and pSyn across conditions and hippocampal subfields. 80–100 µm thick hippocampal sections were immunostained with the AT8 antibody against pTau (Ser202, Thr205) (cyan), the 4G8 antibody against Aβ (AA17-24) (magenta) and the 11A5 antibody against pSyn (Ser129) (green). The stainings show heterogenous distribution and types of inclusions across conditions and hippocampal subregions. Scale bars = 100 µm [file 40478_2022_1342_MOESM6_ESM.pdf]

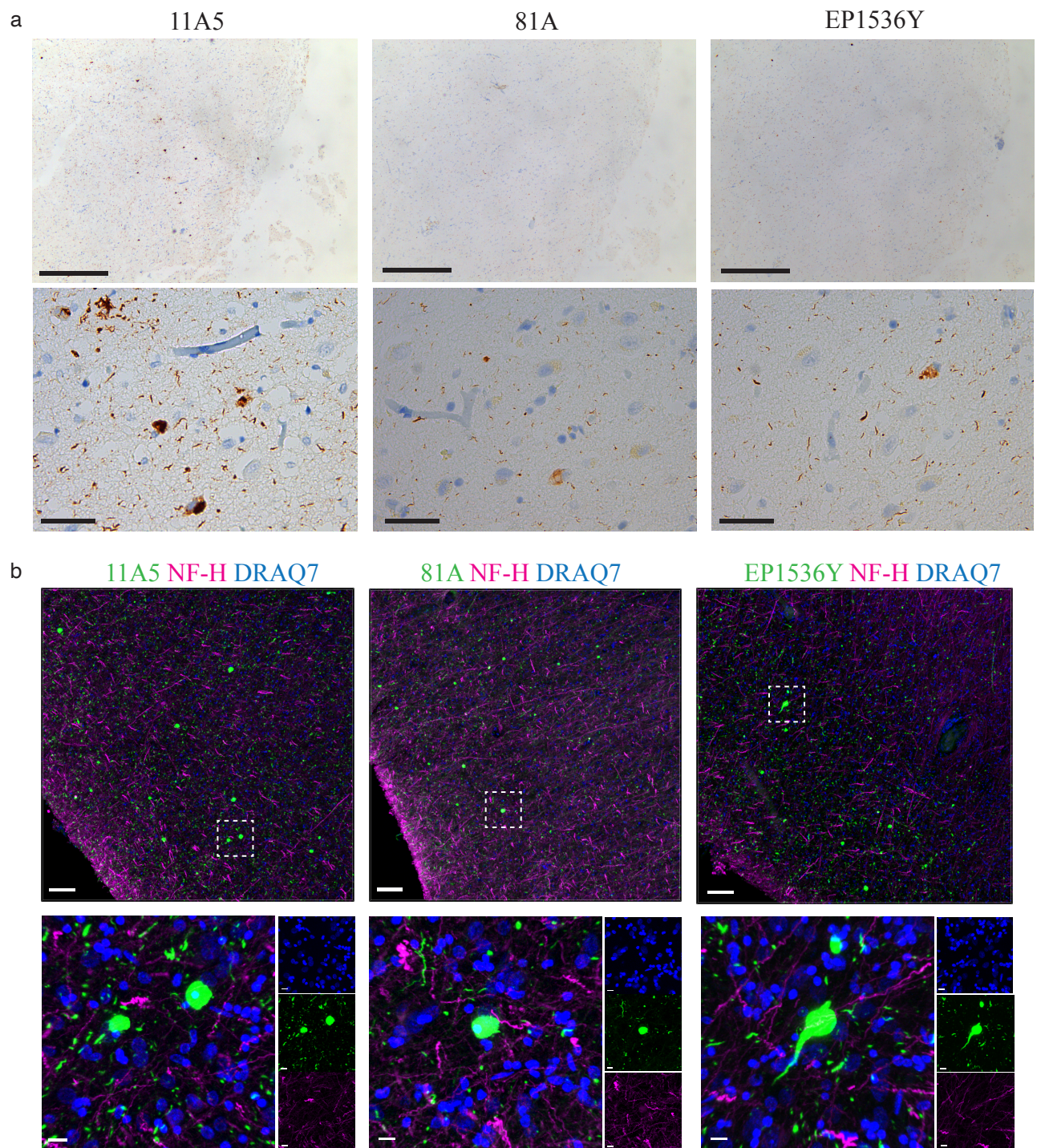

**Supplementary Fig. 7**

Supplement: Supplementary file 7 — Additional file 7: Fig. 7. Validation of pSyn stainings with three different antibodies on FFPE samples of a neuropathologically confirmed DLB case. 3 µm thick paraffin block sections from the amygdala and 80–100 µm thick sections from fixed hippocampus were obtained from the same 91-year-old male DLB patient (case 20) and were stained against pSyn using three different antibodies, namely 11A5, 81A and EP1536Y, that all three recognize the P-Ser-129 epitope. (a) Sections from paraffin blocks were stained against pSyn (11A4, 81A and EP1536Y; brown) based on the DAB/HRP substrate system. The sections were counterstained with hematoxylin. The lower rows represent a zoom of the indicated region in the upper row. (b) Thick sections from fixed samples were stained by immunofluorescence against pSyn (11A4, 81A and EP1536Y; green), neurofilaments (NF-H, magenta) and all nuclei (DRAQ7TM, blue). Scale bars in (a) upper row = 500 µm and lower row = 50 µm; (b) upper row = 100 µm and lower row = 10 µm [file 40478_2022_1342_MOESM7_ESM.pdf]

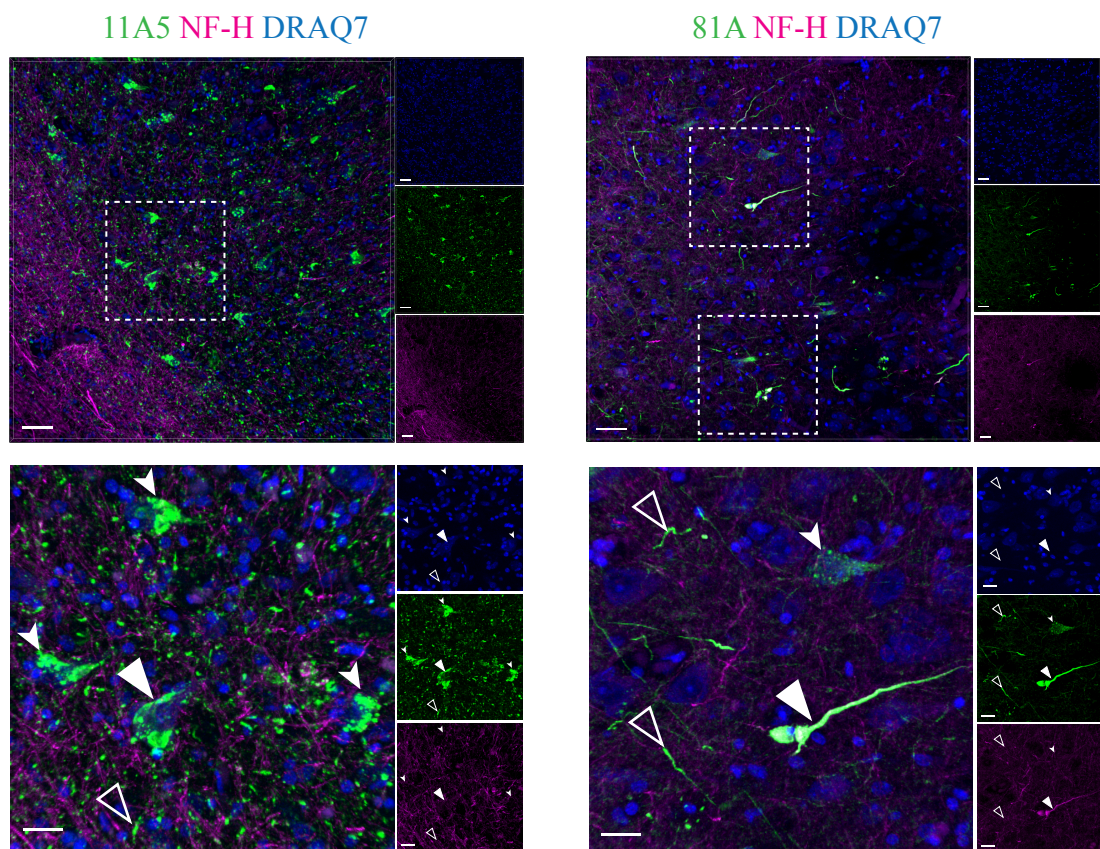

**Supplementary Fig. 8**

Supplement: Supplementary file 8 — Additional file 8: Fig. 8. Confocal description of pSyn staining in a neuropathologically confirmed AD case. 80-100 µm thick sections from fixed hippocampus from a 90-year-old male AD patient (case 28) were immunostained against pSyn (11A5, 81A; green), neurofilaments (NF-H, magenta) and all nuclei (DRAQ7, blue). The upper row shows the hippocampus (stratum oriens at left bottom corner), and the lower row represents a zoom of the marked areas in the pyramidal layer. PSyn inclusions are present under various forms, including PHF-like (full triangle), Lewy neurite (empty triangle) and vacuolar aggregations (arrowhead). Scale bars upper row = 50 µm and lower row = 20 µm [file 40478_2022_1342_MOESM8_ESM.pdf]
