# Supplementary material for: Microglia phenotypes are associated with subregional patterns of concomitant tau, amyloid-β and α-synuclein pathologies in the hippocampus of patients with Alzheimer’s disease and dementia with Lewy bodies
Source: Acta Neuropathol Commun. 2022 Mar 16;10:36. doi: 10.1186/s40478-022-01342-7 (PMC8925098; doi:10.1186/s40478-022-01342-7)
Supplement: Supplementary file 3 — Additional file 3: Fig. S3. List and description of all morphological features with prototypic morphologies for lowest and highest feature values. Scale bars = 20 µm [file 40478_2022_1342_MOESM3_ESM.pdf]

Feature<br>Description	Morphological prototypes<br>Min. value Max. value
**1st largest bound**<br>Largest dimension of bounding box enclosing a structure	
**2nd largest bound**<br>2nd largest dimension of bounding box enclosing a structure	
**1 over 2**<br>Ratio of 1st and 2nd largest bound (elongation)	
**Average curvature**<br>Average curvature of projections (length of edge/distance of nodes)	
**Average node degree**<br>Average degree for all the nodes in the graph	
**Compactness**<br>Ratio between structure volume and volume of convex hull	
**Ending node density**<br>Number of ending nodes over total number of nodes	
**Link density**<br>Number of edges divided by the number of node pairs	
**Max curvature**<br>Maximal curvature of projections (length of edge/distance of nodes)	
**Max edge length**<br>Longest edge in the graph	
**Mean edge length**<br>Mean length of all edges in the graph	
**Node density**<br>Number of nodes divided by total volume	
**Polarity**<br>Average direction of the projections emerging from the central node	
**S-metric**<br>Summed products of nodal degrees across all edges	
**Volume**<br>Number of voxels that constitute the volume	
**Volume / number of edges**<br>Volume in voxels divided by the number of existing edges	

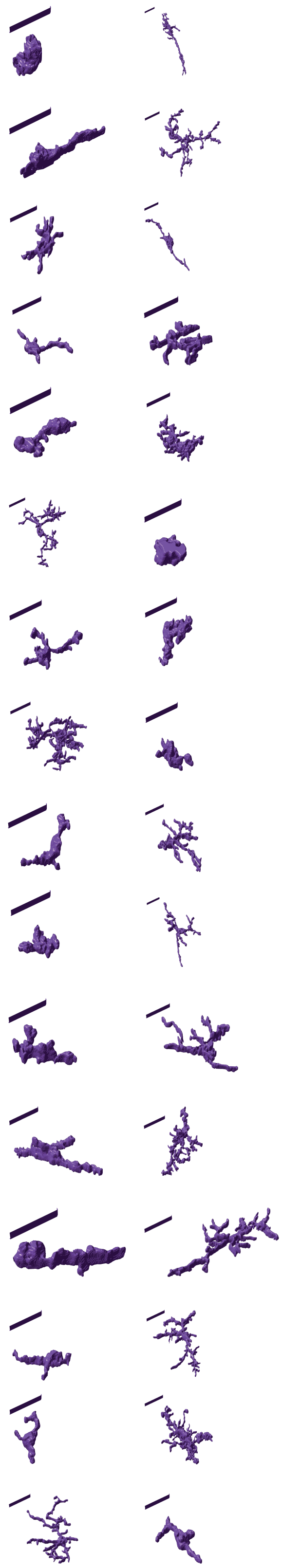

Supplementary Fig. 3
